# Silver Nanoparticle-Deposited Boron Nitride Nanosheets as Fillers for Polymeric Composites with High Thermal Conductivity

**DOI:** 10.1038/srep19394

**Published:** 2016-01-19

**Authors:** Fangfang Wang, Xiaoliang Zeng, Yimin Yao, Rong Sun, Jianbin Xu, Ching-Ping Wong

**Affiliations:** 1 Center for Advanced Materials, Shenzhen Institutes of Advanced Technology, Chinese Academy of Sciences, Shenzhen 518055, China; 2 Department of Nano Science and Technology Institute, University of Science and Technology of China, Suzhou 215123, China; 3 Shenzhen College of Advanced Technology, University of Chinese Academy of Sciences, Shenzhen 518055, China; 4 Department of Electronics Engineering, The Chinese University of Hong Kong, Hong Kong, China; 5 School of Mechanical Engineering, Georgia Institute of Technology, 771 Ferst Drive, Atlanta, Georgia 30332, USA

## Abstract

Polymer composites with high thermal conductivity have recently attracted much attention, along with the rapid development of the electronic devices toward higher speed and performance. However, a common method to enhance polymer thermal conductivity through an addition of high thermally conductive fillers usually cannot provide an expected value, especially for composites requiring electrical insulation. Here, we show that polymeric composites with silver nanoparticle-deposited boron nitride nanosheets as fillers could effectively enhance the thermal conductivity of polymer, thanks to the bridging connections of silver nanoparticles among boron nitride nanosheets. The thermal conductivity of the composite is significantly increased from 1.63 W/m-K for the composite filled with the silver nanoparticle-deposited boron nitride nanosheets to 3.06 W/m-K at the boron nitride nanosheets loading of 25.1 vol %. In addition, the electrically insulating properties of the composite are well preserved. Fitting the measured thermal conductivity of epoxy composite with one physical model indicates that the composite with silver nanoparticle-deposited boron nitride nanosheets outperforms the one with boron nitride nanosheets, owning to the lower thermal contact resistance among boron nitride nanosheets’ interfaces. The finding sheds new light on enhancement of thermal conductivity of the polymeric composites which concurrently require the electrical insulation.

In recent years, heat removal has become a crucial issue for electronic packaging devices, along with their development toward high speed and performance^1^. The need for polymer materials with high thermal conductivity (*K*) has become essential for the design of the next generation of electronic packaging devices^2^,^3^. However, most of the polymers often have a relatively low *K*, ranging from 0.1 to 0.5 W/m-K at room temperature^3^,^4^,^5^,^6^. Various thermally conductive fillers have been embedded into the polymers to improve their thermal conductivities. By averting the high electrical conductivity of the metallic particles, several ceramic materials such as aluminum nitride (AlN)^7^, boron nitride (BN)^8^, silicon carbide (SiC)^9^ and beryllium oxide (BeO) have received significant attention as fillers due to their high *K* values and electrical volume resistivities (*Φ*). Among them, hexagonal boron nitride (*h*-BN), an analog of graphite with layered structures, has demonstrated to be one of the most promising fillers to enhance *K* of polymer, due to its superior thermal and chemical stability, high mechanical strength, and high *K*^10^,^11^,^12^. A two-dimensional (2D) boron nitride nanosheet (BNNS) with an exposed (002) crystal surface would be valuable due to its many unusual properties associated with the ultrathin nature^13^,^14^. Some theoretical studies demonstrated that a *K* value of 1,700–3,000 W/m-K for BNNSs could be obtained^15^,^16^. Many efforts have been made to fabricate polymer composites using BNNSs as nanoscale fillers in polymeric matrices^1^,^17^,^18^. A recent study showed that epoxy nanocomposite filled with BNNSs had a *K* value of 0.83 W/m-K with 30 wt % of BNNSs^1^. However, *K* enhancement was still limited, mainly due to the thermal contact resistance and gaps among fillers. In order to minize the thermal contact resistance and gaps, Jiang *et al*. used BNNSs and α-alumina (α-Al_2_O_3_) as the fillers to increase *K* of the composites^18^. They claimed that the α-Al_2_O_3_ played a bridge role to link the BNNSs together, thus leading to the formation of the effective thermally conductive networks. Nevertheless, the composite only showed the highest *K* of 0.81 W/m-K at 26.5 vol % loading, which is due to the absence of the contacts between α-Al_2_O_3_ and BNNSs. Therefore, it is still technically challenging to fabricate high-K polymer composites filled with BNNSs.

In this work, we design a new type of nanohybrids composed of silver nanoparticles-deposited boron nitride nanosheets (BNNSs/AgNPs) as fillers for the epoxy matrix to achieve a high thermal conductivity (Fig. 1(a)). We have demonsrated that BNNSs/AgNPs can dramatically increase the *K* of an epoxy resin compared with those just containing BNNSs, as shown in Fig. 1(b). We believe that AgNPs with uniform small sizes distributed on the suface of BNNSs could be sintered together in the procecess of epoxy curing and serve as “solders“ to link the individual BNNSs. Therefore the thermal conducting paths are effectively constructed, leading to the decreased thermal contact resistance among BNNSs. The thermal conductivity by 1123% against the pristine expoxy’s one with 25.1 vol % BNNSs content (*V*_*f*_) is obatined, which is useful for the future application as electronic packaging material. In addition, the electrically insulating property of the composite is not compromised.

## Materials and Methods

### Preparation of BNNSs and BNNSs/AgNPs

**Figure 2:**
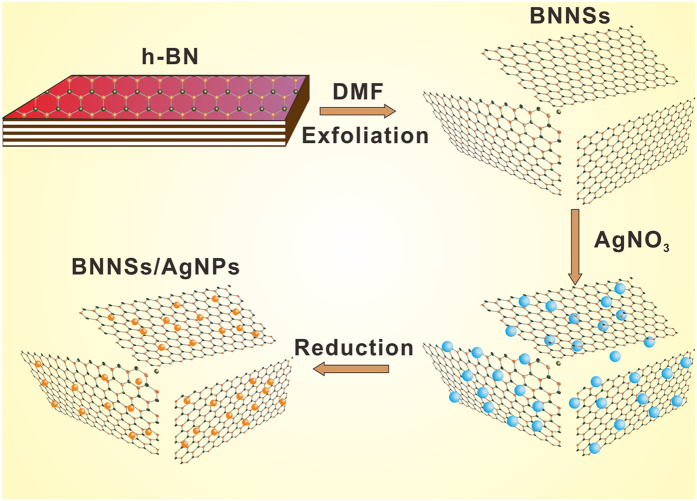
Illustration for the preparation process of BNNS/AgNP nanohybrids.

The BNNSs were prepared using a liquid-phase exfoliation method reported previously^19^,^20^,^21^. In brief, commerical *h*-BN micropowder (2g, 2 μm in size, Denka, Japan) was dispersed in N,N-dimethylformamide DMF (300 ml, purity ≥ 99.5%). The dispersion was sonicated for 48 h in a sonic bath and then centrifuged at 1000 rpm for 20 min. After centrifugation of the dispersions, the supernatant was decanted. Then silver nitrate (AgNO_3_, 0.80 g, 99.8%) aqueous solution (15 mL) was dropped into the exfoliated BNNSs/DMF (2.85 mg/ml, 280 ml) mixture for one hour, at 60 °C with gentle agitation simultaneously. The mixture was then kept without stirring at room temperature for 24 h to allow the AgNPs distribution on the surfaces of BNNSs. DMF acted as both the chemical liquid for exfoliation of *h*-BN and the reductant of Ag^+^^22^,^23^,^24^. The BNNSs/AgNPs/DMF solution was filtered using a polytetrafluoroethylene membrane (0.22 μm) and washed with ethanol and acetone, respectively. The preparation process of BNNSs/AgNPs is shown in Fig. 2.

### Fabrication of epoxy-based composites

Before the addition of BNNSs/AgNPs hybrid, the mixture of liquid crystalline epoxy resin (LCER) 4,4′-Bis(4-hydroxybenzoyloxy)-3,3′,5,5′-tetramethyl(1,1′-bipheyl) (DGE-BHBTMBP, 100%) and the curing agent, 4,4′-diaminodiphenylsulphone (DDS) with a LCER-to-DDS weight ratio of 13:7 was firstly subjected to pre-curing process at 180 °C for 30 min. Then the BNNSs/AgNPs hybrids and the pre-cured epoxy were dispersed in butanone under continuous stirring for 24 hours. The epoxy composite with the homogeneously dispersed BNNSs/AgNPs was bar coated on copper film and cured at the temperature from 150 °C–180 °C, and 220 °C for 2 h, respectively. By cotrolling the content of BNNSs, we prepared a series of composites. For comparison, the composites solely containing BNNSs were prepared in the same way as described above.

### Morphology and structure characterization

Transmission electron microscopy (TEM) micrographs were obtained using JEOL JEM-2100 tranmission electron microscope, operating at 200 keV. X-Ray diffraction (XRD) analyses of the BNNS/AgNP hybrids were recorded at a scan rate of 0.02°/s in the 2θ range of 5–90° using X-ray powder diffractometer with Cu-K radiation. Raman spectra of the BNNS/AgNP hybrids and BNNSs were obtained by utilizing a LabRAM ARAMIS Raman confocal microscope (HORIBA Jobin Y von) with a 514.5 nm laser irradiation. Dispersion process of BNNSs in the epoxy matrix was investigated by examining the fracture morphologies of the composites in scanning electron microscope (SEM,FEI NOVA 4500, FEI). The thermal diffusivities (*α*) of the composites were measured by the laser flash method using LFA 467 (Nano-flash, Netzsch). The thermal conductivity was calculated by *K* = *α* × *C*_*P*_ × *ρ*, in which *C*_*P*_ and *ρ* are the heat capacity and density of the composites, respectively. The *C*_*P*_ was measured using differential scanning calorimetry (DSC, Q-20 TA Instruments). The electric resistance was measrued by Model 6517B electrometer. All the measurements were carried out at room temperature.

## Results and Discussion

### Characterization of BNNS/AgNP by transmission electron microscopy and *x*-ray diffraction

**Figure 3:**
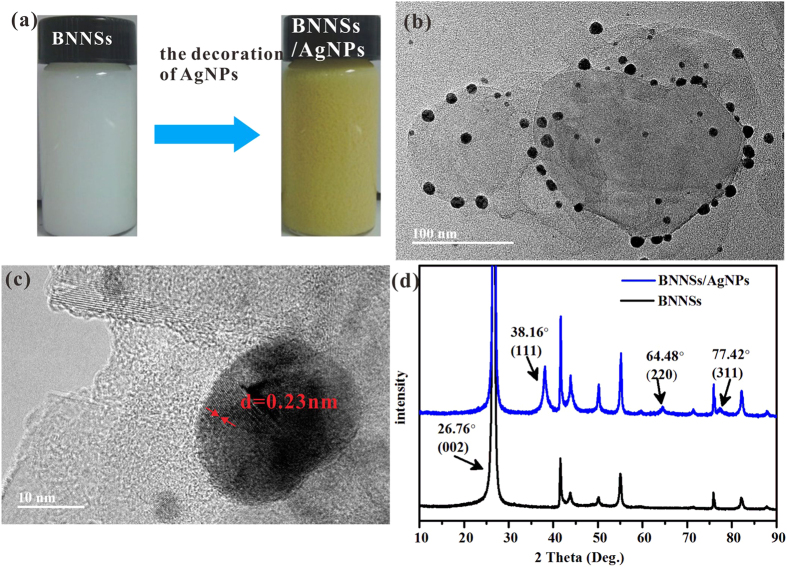
Preparation and characterization of BNNSs/AgNPs hybrids. (**a**) Optical image of BNNSs and BNNSs/AgNPs dispersed in DMF. (**b,c**) TEM micrograph of BNNSs/AgNPs and HRTEM micrograph of AgNPs. (**d**) XRD patterns of BNNSs/AgNPs and BNNSs.

Figure 3(a) presents an optical image of BNNS before and after the decoration of AgNPs. Obviously, the color of BNNS solution turns from “milky” white to yellow. The morphology of BNNSs with the typical lateral size ranging from 100–500 nm and a thickness of 5 nm (less than 15 layers) was determined by TEM (Supplementary Information, Figure S2). Furthermore, the TEM micrograph of the BNNS/AgNP hybrids (Fig. 3(b)) demonstrates that AgNPs with the size of 5 to 20 nm were deposited on BNNSs after the reaction. There do not exist any individual AgNPs in the area beside the BNNS/AgNP hybrids, which indicates that all the formed AgNPs were anchored on the BNNSs. A high resolution TEM micrograph (Fig. 3(c)) shows that the interplanar spacing of the AgNPs lattice is approximately 0.23 nm, which agrees well with the (111) lattice spacing of Ag. The successful decoration of AgNP on BNNS was further confirmed by SEM (Supplementary Information, Figure S3), which agrees well with the TEM results. Figure 3(d) shows the XRD patterns of the BNNSs and the BNNSs/AgNPs. The peaks of BNNSs/AgNPs hybrids at 2*θ* ≈ 27°, 42°, 55°, 76° result from the diffraction of (002), (100), (004) and (110) planes of *h*-BN, which are in good agreemement with those described in the literature^25^,^26^. The new characteristic peaks at 2*θ* ≈ 38°, 64°, 77°, corresonding to the plane (111), (220), (311) of AgNPs^23^,^27^,^28^ can be observed, which further corroborates the decoration of AgNPs on the BNNSs.

### Thermal conductivity of epoxy composites filled with BNNSs/AgNPs and BNNSs

**Figure 4:**
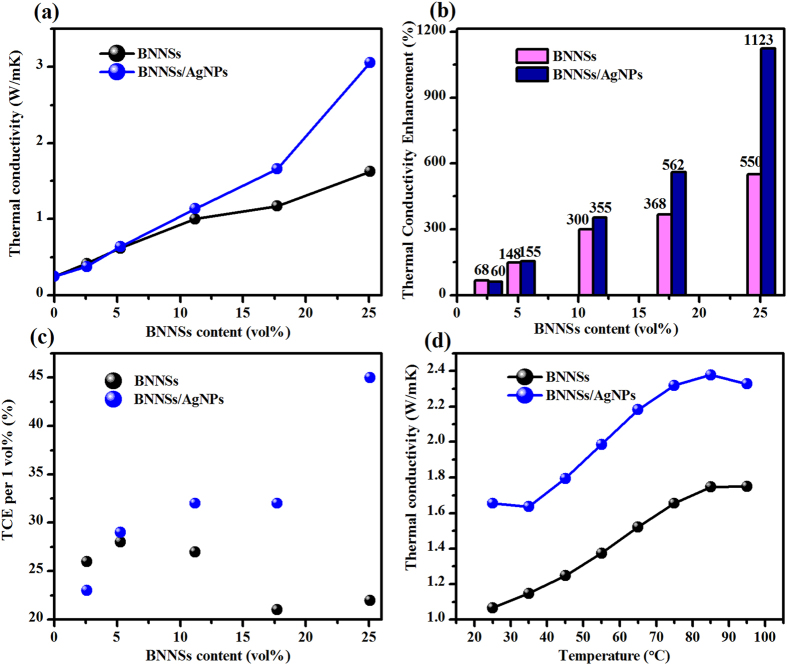
Thermal conductivity of BNNSs/AgNPs/epoxy composite. (**a**) Thermal conductivity of the epoxy composite as a function of BNNSs loading. (**b**) Thermal conductivity enhancement of the composite as a function of BNNSs loading in relation to pristine epoxy. (**c**) Calculated thermal conductivity enhancement of the composite per 1 vol% filler loading. (**d**) Experimentally determined dependence of thermal conductivity of composites on temperature with the BNNSs 17.7 vol% loading.

Figure 4(a) shows *K* as a function of BNNSs content (*V*_*f*_, vol%) for the epoxy composites with or without AgNPs. The pure epoxy has a poor *K* of 0.25 W/m-K, which is in agreement with the previously reported value^29^. After the addition of BNNSs/AgNPs fillers, *K* increases with the increase of *V*_*f*_. It should be noted that there appears no difference in thermal conductivity between the one of epoxy/BNNSs and that of epoxy/BNNSs/AgNPs composites for the BNNSs loading below 11.7 vol%. This is attributed to the fact that the AgNPs cannot function at low filler loading (<11.7 vol%), because the BNNSs/AgNPs hybrids are completely surrounded by the epoxy and no contact exists among fillers. However, the pronounced difference in *K* is observed when the filler loading exceeds 17.7 vol%. However, when the *V*_*f*_ reaches up to 25.1 vol %, it is seen that the *K* of the composite filled with BNNNs/AgNPs hybrids reaches 3.06 W/m-K, while the corresponding *K* for the composite filled only with BNNSs is only 1.66 W/m-K. This suggests that the BNNSs/AgNPs nanohybrids are more efficient to increase *K* than BNNSs alone. The efficiency of the fillers in epoxy matrix can be quantitatively characterized by the thermal conductivity enhancement (*TCE*) defined as ξ = (*K* – *K*_*e*_)/*K*_*e*_, where *K*, and *K*_*e*_ are the thermal conductivities of the composite and the pristine epoxy, respectively. Figure 4(b) shows *TCE* factor as a function of *V*_*f*_ for the epoxy composites with the two different thermal conductive fillers at RT. One can see that the *TCE* factors increase with filler loadings, and the difference between BNNSs and BNNSs/AgNPs is more obvious when the filler loading exceeds 17.7 vol%. For example, the *TCE* of BNNSs/AgNPs/epoxy composite with 2.58 vol % fillers is 60%, while the corresponding value is 68% for BNNSs/epoxy composite. When *V*_*f*_ reaches to 25.1%, the *ξ* value of 1123% can be achieved for BNNSs/AgNPs nanohybrid fillers, a remarkable improvement of *K* compared with BNNSs fillers (*ξ* = 550%). The measured *TCE* per 1 vol % for the composite with BNNSs is about 25% (Fig. 4(c)), slightly higher than that with traditional fillers (~20%)^29^. After the decoration of AgNPs, the *TCE* per 1 vol% of BNNSs/AgNPs/epoxy composite is increased to 45%, which is twice that of BNNSs (*V*_*f*_ = 25.1 vol %). The control experiments with BNNSs show the advantage of BNNSs/AgNPs as an effective thermal conducting fillers to improve the *K* of the composite. This is ascribed to the “bridging” function of AgNPs between two adjacent BNNS species. The AgNPs could be sintered together during the curing process of epoxy, leading to the formation of the thermally conductive networks. Figure 4(d) shows *K* as a function of temperature for *V*_*f*_  = 17.7%. The *K* increases with temperature below 85 °C, and it follows approximately a linear dependence on *V*_*f*_. As the temperature increases successively, *K* does not appear to increase, but there exists a significant plateau in the range of 85 °C. For BNNSs, *K* decreases with increasing temperature, due to stronger phonon Umklapp scatterings^30^. In the presence the pristine epoxy resins, *K* increase with temperature as a result of better phonon transmission through the interfaces and decreased Kapitza resistance^31^. The dual effects result in the observed *K* in the relevant temperature range, which is consisted with the previous report^32^.

### SEM cross section micrographs of the composites with 25.1 vol% BNNSs loading

**Figure 5:**
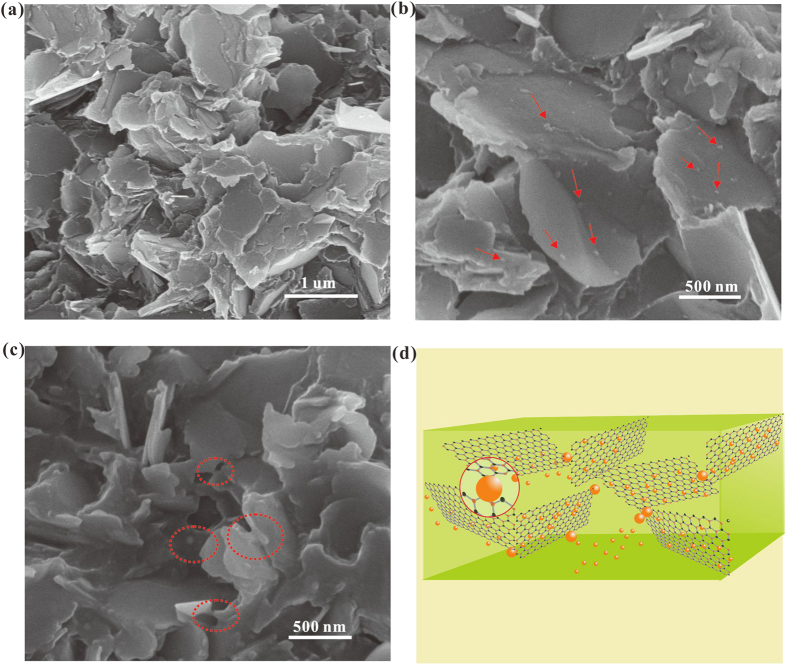
SEM cross section micrographs of the composites with 25.1 vol% BNNSs loading. (**a**) for BNNSs; (**b,c**) for BNNSs/AgNPs). The red arrows in (**b**) show the AgNPs, and the red cycles in (**c**) show the AgNPs bridges among BNNSs. (**d**) a schematic to show how heat to be dissipated between BN layers and AgNPs.

To understand the high *K* enhancement of the composites filled with BNNSs/AgNPs nanohybrids, the representative cross-sectional SEM micrographs of the composites are shown in Fig. 5. The fracture morphology of 25.1 vol % BNNSs/epoxy composite (Fig. 5(a)) shows that there exist much obvious hollow gaps among BNNSs and partial layered structures, which is not tightly stacked in the epoxy matrix. For the fracture morphology of BNNSs/AgNPs/epoxy composite (Fig. 5(b)), several silver nanoparticles are distributed on the surface of BNNSs, whose sizes are larger than that of AgNPs observed by TEM. The result suggests that during the curing progress, small AgNPs merged to form larger AgNPs to decrease surface energy. The curing time and temperature allow the sintering of AgNPs and the epoxy curing to occur simultaneously. The networks are formed through sintering of the particle-particle contacts, as the red circles indicate in the characteristic regions (Fig. 5(c)). As illustrated in Fig. 5(d), the AgNPs attached on the surfaces of BNNSs play a role of bridging the nanosheets together. Due to the large contact areas between BNNSs, the thermally conductive networks are easily formed with the extended heat transfer pathways through BNNSs/AgNPs/epoxy composites.

### Measured and simulated thermal conductivities of epoxy composites with a physical model

**Figure 6:**
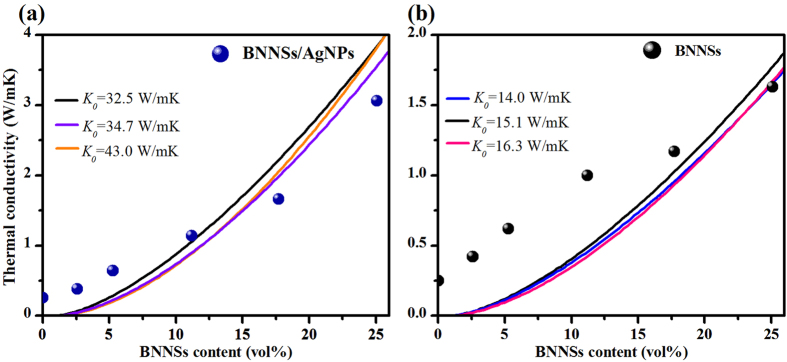
Fitting of the theoretical curves to the experimental data is conducted for extraction of the effective thermal conductivity of filler networks and thermal contact resistance among fillers. Measured and simulated thermal conductivities of (**a**) BNNSs/AgNPs/epoxy composite, and (**b**) BNNSs/epoxy composite as a function of filler content.

Many research groups have computationally simulated the thermal boundaries within nanofillers in order to decrease the thermal contact resistance and Umklapp phonon scatterings^33^,^34^,^35^,^36^. Though numerous research activities have been conducted on thermally conductive fillers to date, heat flow at fillers’ interfaces still remains a subject of fundamental challenges. Here, a physical model proposed by Foygel *et al*.^35^ is applied to our experimental values of *K* for BNNSs/epoxy and BNNSs/AgNPs/epoxy composites. This model is based on fillers’ random distribution and percolating networks in matrix. The geometry of the junction formed among BNNSs is emphasized to analyze the nanoscale interfacial thermal physics across two BNNS species bridged by AgNPs. The thermal conductivity of epoxy composites can be described as the following function of *V*_*f*_, which is represented by Equation (1).


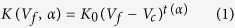


where *K*_0_ is a pre-exponential factor depending on the thermal conductivity of the contacting fillers or the effective thermal conductivity of the filler networks. *V*_*c*_ is the critical volume fraction at the thermal percolation threshold, and *t*(*α*) is a conductivity exponent dependent on the aspect ratio (*α*) of BNNSs. According to Equation (S1) (Supplementary Information), the value of *V*_*c*_ (0.01) was obtained. Then we use Equation (1) to fit the experimental data with fitting parameters *K*_0_ and *t*(*α*), as shown in Fig. 6(a). For BNNSs/AgNPs/epoxy composites, the pre-exponential factor *K*_0_ in Equation (1) can be estimated as *K*_0_ = 32.5–43.0 W/m-K. The fit follows most of the experimental points, especially in high filler loadings. The thermal contact resistance, *R*_*c*_ between fillers is defined as


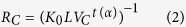


The value for *R*_*c*_ obtained from Equation (2) is (3–5) × 10^8^ K/W. As a control, for BNNSs/epoxy, the effective thermal conductivity *K*_0_ of the BNNSs networks is 14–16.3 W/m-K, as shown in Fig. 6(b). Then Equation (2) yields a *R*_*c*_ = (7–9) × 10^8^ K/W for BNNSs/epoxy composites, greater than BNNSs/AgNPs. Therefore, the results suggest that the thermal transport between BNNSs has been significantly altered, after the decoration of AgNPs on the surfaces of BNNSs. The thermal contact resistance between BNNSs is shown to decrease with the connections by AgNPs, resulting in the highly effective thermal networks. The interfacial thermal contact resistance is substantially minimized, and therefore a larger *K* can be obtained in BNNSs/AgNPs/epoxy composite.

### Thermal conductivity values for composites with various fillers

Table 1 show that the measured *K* of BNNSs/AgNPs/epoxy composites is indeed high compared with others. We attribute it to the fact that the BNNSs form the major thermally conductive pathways in the composites and the AgNPs bridge the connections among BNNSs.

### Electrical conductivity at different BNNSs contents in epoxy composites

**Figure 7:**
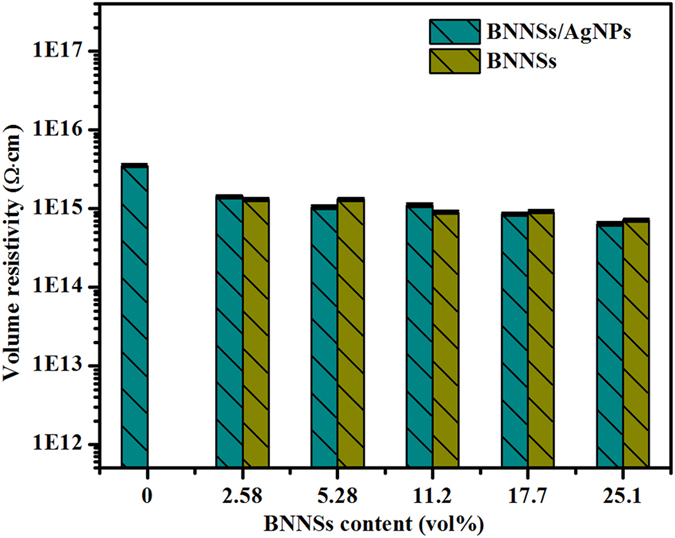
Electrical conductivity at different BNNSs contents in epoxy composites. (**a**) BNNSs/AgNPs/epoxy. (**b**) BNNSs/epoxy.

As we all known, BNNSs is an electrical insulator^43^ and considered as the promising dielectric material in the most applications of graphene based electronic devices. Therefore, we anticipate that the high *K* of the composites after the decoration of AgNPs will not be compromised by decreased volume resistivity. Figure 7 shows the volume resistivity (*Φ*) of the composites as a function of *V*_*f*_. All the composites containing the two kinds of thermally conductive fillers present a characteristic of the insulator (*Φ* > 10^9^ Ω·cm), indicating the increase in *K* without substantial change in *Φ*. In our experiment, the AgNPs are speculated to be mainly attached onto the defect sites and the active edges of BNNSs covered with some functional groups such as hydroxyl groups (−OH) and amino groups (−NH_2_)^44^,^45^. The AgNPs may be not enough to increase the electrical conductivity. Often, the formation of the percolation network facilitates a decrease in *Φ* of the composite with the electrically insulating matrix. However, the percolation networks in the composite are not formed completely. The heat can be dissipated through phonon transmission in BNNSs, whereas the electronic transport through AgNPs cannot fully occur. On the other hand, the electrically insulating matrix and BNNSs may form the tunneling barrier for the electrons and effectively eliminate the electrical transport.

## Conclusions

In summary, benefiting from the bridging connections of AgNPs among BNNSs formed during the curing process of the epoxy, a new kind of thermally conductive and electrically insulating epoxy composites are produced. The thermal conductivity of the BNNSs/AgNPs/epoxy composite increases with the filler content, and reaches its highest value of 3.06 W/m-K, approximately twice as high as the counterpart of BNNSs/epoxy composite. The simulated results demonstrate that the remarkable improvement is due to the decreased thermal resistance between BNNSs with the bridging connections by AgNPs, rendering the highly effective thermal networks. Our findings contribute to the mechanistic understanding of the bridging connections among the fillers for the thermal properties of epoxy composites. The BNNSs/AgNPs/epoxy composites pave the way for their applications in advanced electronic packaging technology, namely thermal interface materials, underfill materials, molding compounds, and flexible substrates.

## Additional Information

**How to cite this article**: Wang, F. *et al*. Silver Nanoparticle-Deposited Boron Nitride Nanosheets as Fillers for Polymeric Composites with High Thermal Conductivity. *Sci. Rep*. **6**, 19394; doi: 10.1038/srep19394 (2016).

## Supplementary Material

Supplementary Information

## Figures and Tables

**Figure 1:**
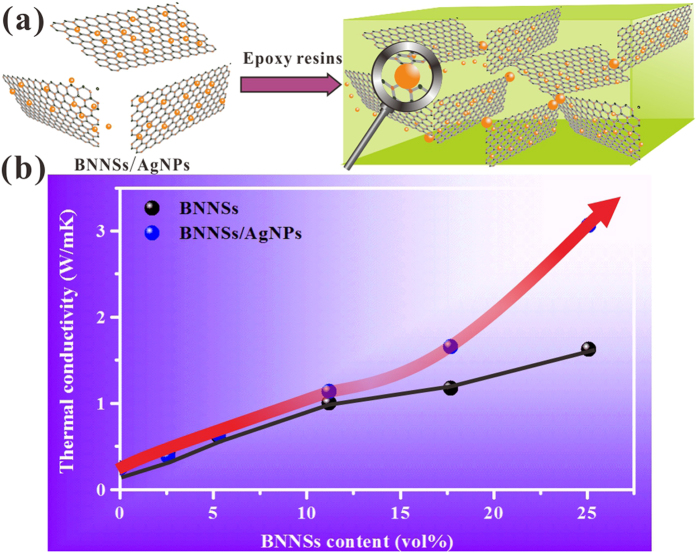
A fabrication route for epoxy composites filled with boron nitride nanosheets decorated with silver nanoparticles and their corresponding thermal conductivities.

**Table 1:** Thermal conductivity values of polymeric composites with different BN-based fillers.

Filler	K (W/m K)	TCE	fraction	matrix	Reference
BNNSs	0.13	51%	5 vol%	polysiloxane	37
h-BN microplates	0.44		5.9 vol%	polyvinyl alcohol	8
BNNSs	0.62	316%	30 wt%	epoxy	1
BNNTs/BNNSs	0.47	147%	2 wt%	epoxy	38
BNNTs	0.50	194%	10 wt%	polymethylmethacrylate	39
micro-and nano-BN	1.2	500%	30 wt%	polyimide	40
BN/Al_2_O_3_	0.81	326%	26.5vol%	epoxy	18
micro+nano-BN	0.60	275%	20wt%	epoxy	41
BN nanosphere	1.10	1375%	67.6vol%	polystyrene	42
BNNSs/AgNPs	3.05	1123%	25.1vol%	epoxy	This work
