# Supplementary material for: Silver Nanoparticle-Deposited Boron Nitride Nanosheets as Fillers for Polymeric Composites with High Thermal Conductivity
Source: Sci Rep. 2016 Jan 19;6:19394. doi: 10.1038/srep19394 (PMC4726004; doi:10.1038/srep19394)
Supplement: Supplementary Information [file srep19394-s1.pdf]

Supplementary Information

Silver Nanoparticle-Deposited Boron Nitride Nanosheets as Fillers for Polymeric Composites with High Thermal Conductivity

Fangfang Wang[1,2], Xiaoliang Zeng[1,3,*], YiminYao[1,3], Rong Sun[1,*], Jianbin Xu[4,*], and Ching-Ping Wong[4,5]

[1]Center for Advanced Materials, Shenzhen Institutes of Advanced Technology, Chinese Academy of Sciences, Shenzhen 518055, China.

[2]Department of Nano Science and Technology Institute, University of Science and Technology of China, Suzhou 215123, China.

[3]Shenzhen College of Advanced Technology, University of Chinese Academy of Sciences, Shenzhen 518055, China.

[4]Department of Electronics Engineering, The Chinese University of Hong Kong, Hong Kong, China.

[5]School of Mechanical Engineering, Georgia Institute of Technology, 771 Ferst Drive, Atlanta, Georgia 30332, USA.

***e-mail: xl.zeng@siat.ac.cn; rong.sun@siat.ac.cn; jbxu@ee.cuhk.edu**

Supplementary Figure S1

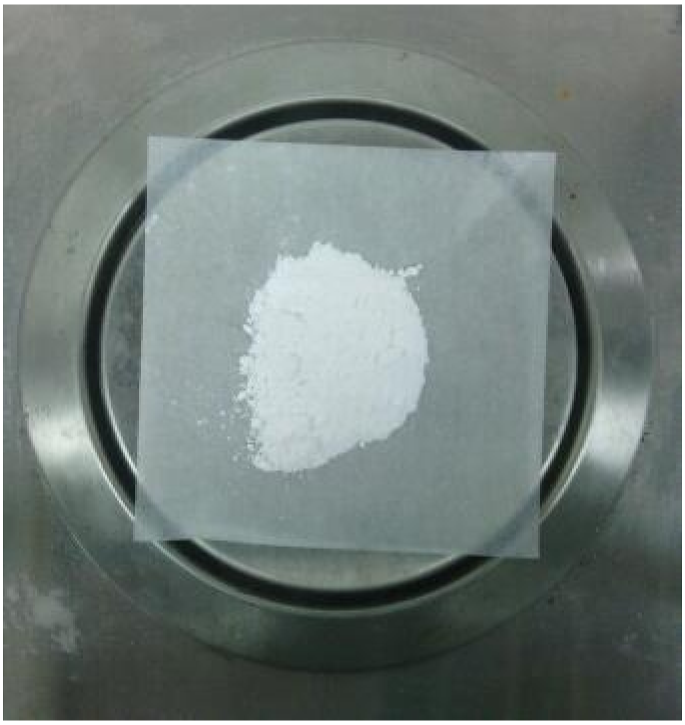

Figure S1 Optical image of h-BN (2 μm).

Supplementary Figure S2

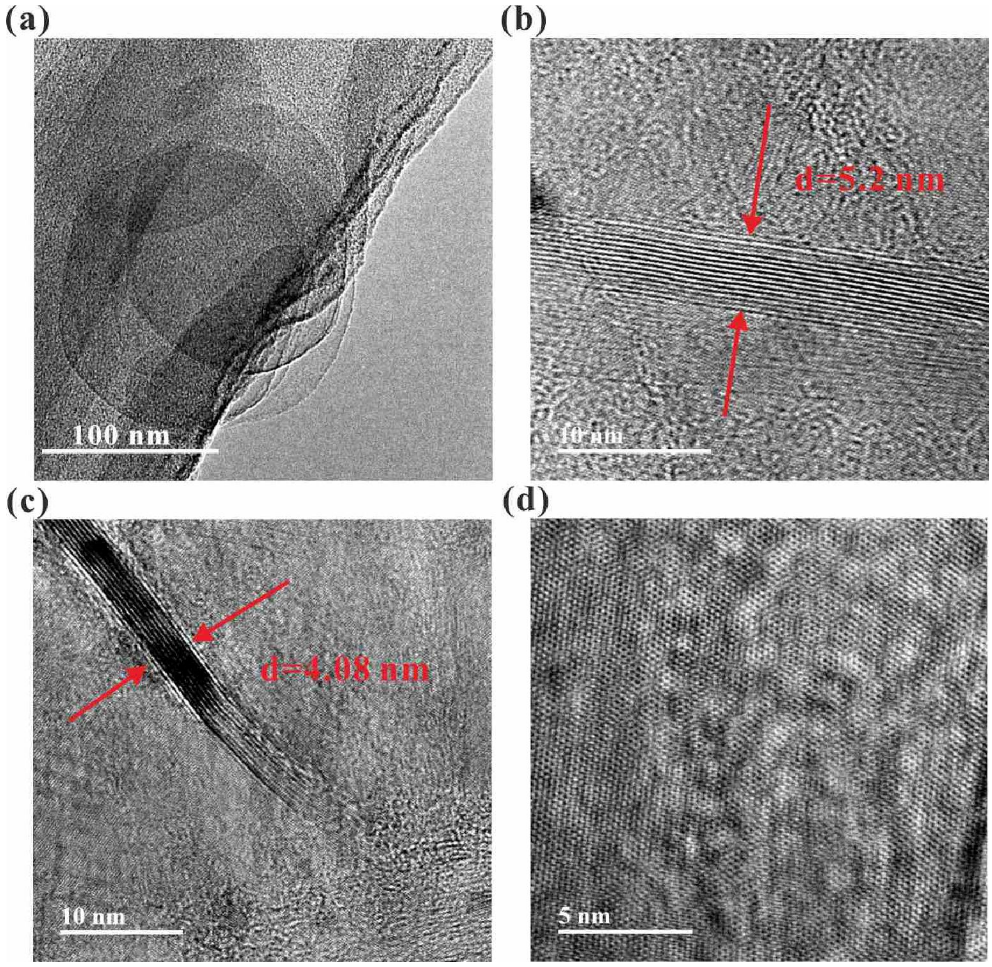

Figure S2. (a) TEM image of BNNSs; (b) and (c) TEM images showing the edge of BN nanosheets. (d) High resolution TEM image to show the crystalline pattern of h-BN.

Supplementary Figure S3

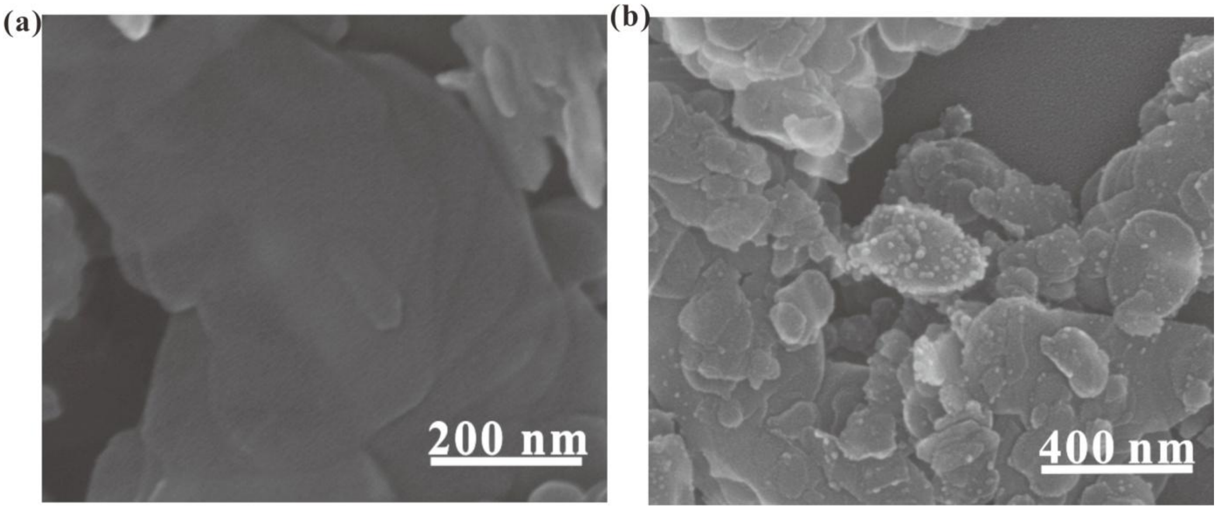

Figure S3. SEM images of BNNSs (a) and BNNSs/AgNPs (b).

Supplementary Figure S4

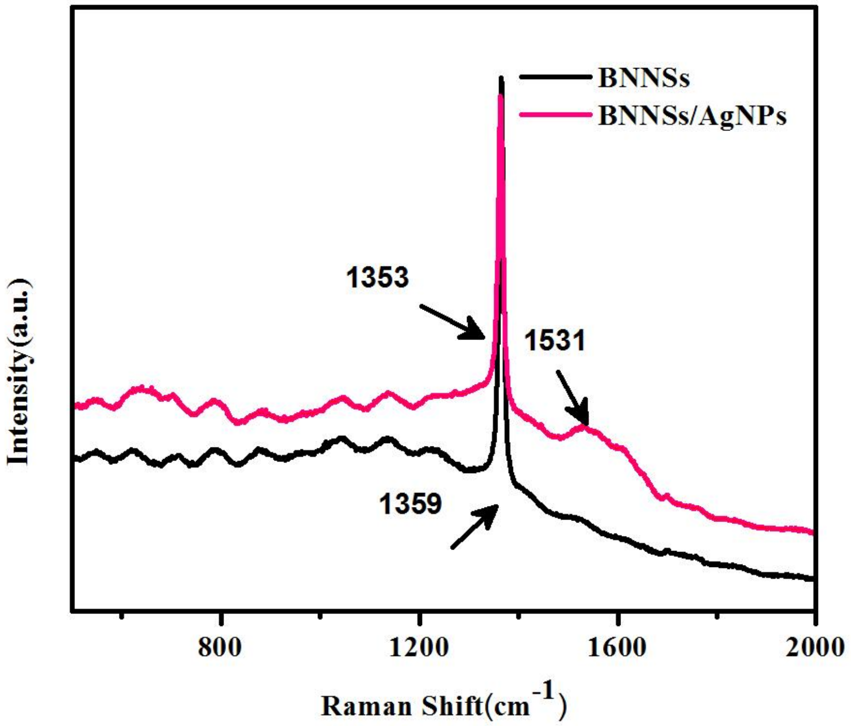

Figure S4 Typical Raman spectrum of BNNSs and BNNSs/AgNPs.

To further investigate the presence of the AgNPs in the as-abtained BNNSs/AgNPs, the typical Raman spectra were taken from the BNNSs and the BNNSs/AgNPs. A dominant peak near 1359 $cm^{-1}$of the BNNSs can be found in the spectrum, which is close to the characteristic Raman peak of bulk BN materials[1, 2]. The peak could be attributed to the B-N vibrational mode (E_{2g}) within h-BN layers, showing the hexagonal structure of the prepared BNNSs[1-5]. Besides, a slight frequency shift (about 6 cm^{-1}) toward a lower wavenumber of the band of BNNSs is found in BNNSs/AgNPs compared with BNNSs. While the BNNSs/AgNPs shows single the Raman peak at 1531 cm^{-1} , which might come from the interaction between AgNPs and the surface functional groups of BNNSs. These results further support the conclusion that AgNPs have been effectively assembled on BNNSs.

Theoretical Approach Used in the Data Analysis

Figure S2 shows the size of BNNSs of mean thickness (*H*) 3.4 nm (=h×*n*, where h=0.34 nm is the thickness of the single-layer BNNS)and length (*L*) 200 nm. The average aspect ratio α=59 (≈*L*/*H*). Using Foygel' results and Monte Carlo simulations, for large $\alpha \gg 1$, the V_c is inversely proportional to α.

$$Vc(\alpha \gg 1) \approx \frac{0.6\ \ 0}{\alpha} \qquad \text{(S1)}$$

Then V_c=0.01.

References

1. Nemanich, R.J., Solin, S.A. & Martin, R.M. Light scattering study of boron nitride microcrystals. *Physical Review B* **23**, 6348-6356 (1981).
2. Hoffman, D.M., Doll, G.L. & Eklund, P.C. Optical properties of pyrolytic boron nitride in the energy range 0.05—10 eV. *Physical Review B* **30**, 6051-6056 (1984).
3. Geick, R., Perry, C.H. & Rupprecht, G. Normal Modes in Hexagonal Boron Nitride. *Physical Review* **146**, 543-547 (1966).
4. Que, R. et al. Generating electric current based on the solvent-dependent charging effects of defective boron nitride nanosheets. *ACS Appl Mater Interfaces* **6**, 19752-19757 (2014).
5. Yu, J. et al. Vertically aligned boron nitride nanosheets: chemical vapor synthesis, ultraviolet light emission, and superhydrophobicity. *ACS Nano* **4**, 414-422 (2010).
